# The Special Issue on “The Nutritional Value of Pulses and Whole Grains”: A Continued Endeavor to Delineate Their Benefits for Today and Addressing the Challenges of the Future

**DOI:** 10.3390/nu14163381

**Published:** 2022-08-17

**Authors:** Christopher P. F. Marinangeli

**Affiliations:** Regulatory Centre of Excellence, Protein Industries Canada, 200-1965 Broad Street, Regina, SK S4P 1Y1, Canada; christopher@proteinsupercluster.ca; Tel.: +1-905-330-0514

Dietary patterns are increasingly focusing on the interplay between nutritional adequacy, reduction of chronic disease, and environmental sustainability. While both pulses and whole grains have a rich history as part of healthy and sustainable dietary patterns [1,2], there is ongoing interest in the use of these foods, and their ingredient derivatives, to delineate effects on multiple aspects of human health and quantify their individual and societal benefits. Both pulses and whole grains are considered to be nutrient dense foods, with fibre and micronutrients being common nutritional attributes that are promoted in dietary guidelines [3]. Pulses contain considerably higher levels of protein compared to whole grains [3]. However, amino acid complementarity between these foods is an additional value proposition, as pulses are leveraged in diets that index higher on plant protein sources and can be an efficient means of replacing animal-derived proteins with those from plants [4].

This Special Edition of Nutrients, “The Nutritional Value of Pulses and Whole Grains” provides a series of papers that touch on various topics and themes that are relevant to a changing food landscape aimed at incorporating more pulses and whole grains into diets. In addition to identifying near and future benefits of these foods, the provided analysis underscores some of the underlying challenges around their incorporation into diets and examination of benefits, which could be critical for using whole grains and pulses in a manner that aligns with global dietary objectives.

Whole grains and pulses are a common thread in healthy dietary patterns. Whether emphasized by specific dietary guidelines in a jurisdiction, as a pattern of eating based on shared attributes across a region, such as the Mediterranean diet, or to tackle societal challenges across metrics of health and sustainability, both pulses and whole grains are touted for their nutritional contributions. Low consumption of whole grains and pulses (and other legumes) are associated with well over 3 million deaths, primarily due to cardiovascular disease [5]. On its own, diets low in dietary fibre have been associated with ≥1million deaths from cardiovascular disease and diabetes and ≥20 million disability-adjusted life years globally [5]. Studies have reviewed the effects of pulses and whole grains on reducing risk factors for cardiometabolic diseases, such as lipids, blood pressure, and glycemic response [6,7,8]. Two reviews published as part of this compendium offer an update and summation of data linking whole grain and pulse (lentil) on markers of inflammation and post-prandial glycemic response, respectively.

The review by Milesi et al. [9] provides a systematic assessment of whole grain consumption on inflammatory biomarkers using criteria that aligns with an accepted definition of whole grains in adults. Analysis of 31 randomized clinical trials (RCTs) showed that overweight/obese individuals and those with pre-existing health conditions demonstrated a reduction in markers of inflammation, primarily CRP [9]. The study by Clark et al. [10] showed that at least 110 g lentils is required to generate a relative reduction in post-prandial glycemic response by 20%, with effects most strongly correlated with levels of protein (r = 0.5513) and fibre (r = 0.3326). Low glycemic response and glycemic index foods have been promoted for reducing risk of cardiometabolic diseases and diabetes management [11,12,13,14]. Using the diabetic rat model, the study by Ren et al. [15] investigated mechanisms for hypoglycemic effects of foxtail millet, a cereal grain that is cultivated across 26 countries. 16S RNA sequencing revealed a correlation between abundance of Lactobaccillus and Ruminococcus_2 and lower fasting and post-prandial glycemic levels. Molecular analysis demonstrated activation of the P13K/AKT signaling pathway, leading to decreased gluconeogenesis and increased glycolysis; and inflammation was suggested given the observed down regulation of NFκB. Collectively, these studies strengthen the importance of carbohydrate quality in food choices, which encompasses “whole food” constituents, low glycemic response and glycemic index foods, and dietary fibre [16]. Carbohydrate quality is increasingly emphasized as a value proposition for consumers to choose foods with significant effects on dietary quality and reduced risk factors for chronic disease [17,18]. The information disseminated in this Special Issue brings new perspectives and data to support the use of pulses and whole grains as healthy carbohydrate foods.

As briefly discussed above, the global burden of disease reports have been effective at translating the societal costs of unhealthy dietary patterns. Over the last decade, additional analyses have aimed to assign a “cost-of-illness” as a novel perspective by evaluating potential healthcare savings when a proportion of a population adopts a healthy diet. In the past, various diets [19], and their components, including, pulses, and whole grains have been associated with significant direct and indirect healthcare cost savings by estimating putative associations with constipation, cardiovascular disease, and cancer in Canada [20,21] and the US [22,23]. In this issue, this analysis was expanded to Australia and Finland. Abdullah et al. [24] showed that increasing whole grain consumption to a 48 g/day target across 5 to 100% of the population could decrease healthcare costs associated total and colorectal cancer by 126.2 M to 1.37 B AUD over 20 years. Similarly, Martikainen et al. [25] used 3 theoretical scenarios (1. 10% unit increase in the Finnish population consuming at least one whole grain serving per day; 2. Increase consumption of one or more whole grain food servings per day among adults already consuming at least one whole grain serving per day; 3 a combination of scenarios 1 and 2) for increasing servings whole grains to estimate potential reductions healthcare costs associated with type 2 diabetes in Finland [25]. Despite already high consumption rates of whole grains compared to other countries in the EU, 286 M€ to 989 M€ in healthcare and productivity cost savings were projected over 10 years, respectively, across scenarios 1–3. Over 30 years, modeled savings increased from over 1.2 B€ to 4.2 B€ and generated 44,237 to 154,094 quality-adjusted life years [25]. In addition to better health, these and other data support top-down dietary guidelines and policies in the context of a balanced and healthy diet to drive broad societal benefits.

One cannot ignore current and future challenges for expanding consumption of both pulses and whole grains. While promoted in dietary guidelines, consumption of these two foods remains relatively low relative to recommendations [1]. The analysis of 6 cycles of NHANES by Mitchell et al. [26] from this collection demonstrated no significant trend in pulse consumption from 2003–2014 with per capita consumption ranging between 19.3 and 24.9 g/day. Although pulse consumers reported higher intakes of dietary fibre, folate, potassium, iron, and protein at intakes ≥69.4 g/day compared to non-pulse consumers, only 27% of adults consumed pulses on one of the two days of the survey [26]. These low consumption rates of pulses mirror previous analysis of the NHANES [27] and the Canadian Community Health Survey [28]. This is corroborated by other data demonstrating that pulses are relatively minor contributors to total protein intakes of diets in Canada [29], the US (~1.3%) [30], France (<1%) [31,32], and the UK (not reported as a significant source of protein) [33]. While consumers are somewhat more familiar with whole grains, in some regions, such as the US, only a fraction of the recommended level of intake have been shown to be consumed on any given day [34].

Over the last decade however, there has been significant growth in the number of manufactured food products that are leveraging whole grains and pulses as ingredients to bolster their actual or perceived nutritional density. The study by Bielefeld et al. [35] demonstrated that the number of legume food products grew from 312 products in 2019 to 610 in 2021 across four major grocery retail outlets in Sydney Australia. Furthermore, legume-formulated snack foods showed the greatest increase (n = 88), with the legume chip category growing by 357%. Nutrient content claims represented the most prominent type of claim used across foods, with most claims promoting foods as a source of dietary fibre (n = 246), gluten free (n = 216), and source of protein (n = 208) [35]. Claims identifying legume foods as clean label (no artificial colours, flavours or preservatives) (n = 252), vegetarian/vegan (n = 232), and organic (n = 115) were also prominent in the 2021 food audit. However, the analysis of nutritional quality of whole grain cereal-based products within the Italian retail market suggested that, without a harmonized legal definition of whole grains, the presence of whole grains cannot be used as a stand alone marker of nutritional quality [36]. Levels of dietary fibre were similar between foods formulated entirely or partially with whole grain ingredients, with these foods containing more sodium than refined grain products. These results speak to some of the consumer challenges in food innovation, with varying motivations and priorities of food attributes across consumer segments [36]. In the same regard, the study by Sajdakowska et al. [37] evaluated consumer motivations and perceptions of pasta and pasta with added fibre. Results segmented consumers as quality, sensory, convenience, or neutral-oriented, with health and fibre promoting statements scoring highest in the quality-oriented segment compared to other groups. Sensory-oriented individuals also indicated that pasta with added fibre has a less appealing taste and visual appearance compared to other groups. These results align with other consumer analysis where taste and price have been the top two purchase drivers in the US over the last 10 years [38]. Overall results corroborate various initiatives to enhance the nutritional profile of manufactured foods, where pulses and whole grains, can underpin such efforts. However, deliverance on those functional drivers will be required for these foods to support adoption of healthy dietary patterns, but likely cannot be achieved without understanding consumer attitudes and motivations toward food choices.

Finally, the final two articles target some of the ongoing challenges for facilitating and understanding the impacts of whole grains and pulses on dietary patterns. The first was led by members of the Whole Grain Initiative; a global consortium comprised of members of academia, government, and industry with a focus on promoting whole grain consumption [39]. The consortium stresses that lack of consensus on a definition of whole grains, and a whole grain food, creates inconsistencies for consumers achieving evidenced-based benefits from whole grain consumption [39]. Recall, that using an acceptable definition of whole grain foods was used by Melesi et al. [9] to summarize the association between whole grain intake and markers of systemic inflammation and the study by Dall’Asta et al. [36] suggested that not having a legal definition of whole grains has created a heterogeneous food environment in Italy with inconsistent nutritional attributes around whole grain foods. In this regard, van der Kamp et al. [39] suggest that only foods containing a minimum of 25% whole grain ingredients (based on dry weight) be eligible for a front-of-pack whole grain claim. Given the breadth of experts establishing proposed definitions for whole grain foods, the proposed definitions and labelling requirements could be used by regulatory agencies for the development of nutritional policies.

The remaining paper by Mitchell et al. [40] discusses the limited data that is available for evaluating intakes of pulses and pulse-derived ingredients. As mentioned previously, consumption levels of pulses are low in many developed jurisdictions that acquire populational food intake data through national surveys. However, given that pulses are legumes, but not all legumes are pulses, pulse consumption is often measured as part of a broader legume food group [40]. This presents some challenges, as pulses have unique nutritional attributes and patterns of consumption compared to other legumes, such as soy and peanuts. The editorial by Mitchell et al. [40] highlights this challenge in the context of the US, where, not until the 2020–2025 Dietary Guidelines for Americans” had the term “pulse” been recognized. With a global focus on enhancing consumption of pulses as part of healthy and sustainable dietary patterns, using the specific terminology to identify pulses in epidemiologic databases is required for generating optimal consumption rates and to create more robust data sets regarding the effects of pulses on nutrient intakes and chronic disease outcomes.

This Special Issue of Nutrients provides a snapshot of interesting developments in the value of pulses and whole grains in healthy dietary patterns. These foods and their ingredients can be useful for bolstering the nutritional value of manufactured food products. However, understanding the expectations of consumers could be critical for offering foods that deliver on individual and societal benefits. At the same time, a judicious examination of policies, regulations, and research methods could be a meaningful exercise to further delineate and ascertain benefits from using these foods more liberally in the food system. Whole grains and pulses continue to be dietary assets that align with global dietary objectives across various nutrition, health, and economic goals.

## Data Availability

Not applicable.
